# Environmental Factors Determining the Epidemiology and Population Genetic Structure of the *Bacillus cereus* Group in the Field

**DOI:** 10.1371/journal.ppat.1000905

**Published:** 2010-05-20

**Authors:** Ben Raymond, Kelly L. Wyres, Samuel K. Sheppard, Richard J. Ellis, Michael B. Bonsall

**Affiliations:** 1 School of Biological Sciences, Royal Holloway University of London, Egham, Surrey, United Kingdom; 2 Department of Zoology, University of Oxford, Oxford, United Kingdom; 3 Molecular Pathogenesis and Genetics, Veterinary Laboratories Agency, Addlestone, United Kingdom; University of Toronto, Canada

## Abstract

*Bacillus thuringiensis* (*Bt*) and its insecticidal toxins are widely exploited in microbial biopesticides and genetically modified crops. Its population biology is, however, poorly understood. Important issues for the safe, sustainable exploitation of *Bt* include understanding how selection maintains expression of insecticidal toxins in nature, whether entomopathogenic *Bt* is ecologically distinct from related human pathogens in the *Bacillus cereus* group, and how the use of microbial pesticides alters natural bacterial populations. We addressed these questions with a MLST scheme applied to a field experiment in which we excluded/added insect hosts and microbial pesticides in a factorial design. The presence of insects increased the density of *Bt/B. cereus* in the soil and the proportion of strains expressing insecticidal toxins. We found a near-epidemic population structure dominated by a single entomopathogenic genotype (ST8) in sprayed and unsprayed enclosures. Biopesticidal ST8 proliferated in hosts after spraying but was also found naturally associated with leaves more than any other genotype. In an independent experiment several ST8 isolates proved better than a range of non-pathogenic STs at endophytic and epiphytic colonization of seedlings from soil. This is the first experimental demonstration of *Bt* behaving as a specialized insect pathogen in the field. These data provide a basis for understanding both *Bt* ecology and the influence of anthropogenic factors on *Bt* populations. This natural population of *Bt* showed habitat associations and a population structure that differed markedly from previous MLST studies of less ecologically coherent *B. cereus* sample collections. The host-specific adaptations of ST8, its close association with its toxin plasmid and its high prevalence within its clade are analogous to the biology of *Bacillus anthracis*. This prevalence also suggests that selection for resistance to the insecticidal toxins of ST8 will have been stronger than for other toxin classes.

## Author Summary

*Bacillus thuringiensis* is one of the most useful bacteria in insect pest management: it is used as an environmentally friendly biopesticide and its insecticidal toxins are incorporated into genetically modified crops. Concerns for its ongoing economic exploitation include the rapidity with which insects can evolve resistance to its toxins and whether *B. thuringiensis* is ecologically and genetically distinct from closely related strains that cause infections in humans and domestic animals. We found that natural bacterial populations in soil and on cabbage leaves were dominated by an insect-pathogen specialist genotype, and this genotype was better than its close relatives at establishing populations on leaves where its hosts were likely to be feeding. Spraying microbial pesticides and the addition of insect hosts increased the proportion of insect-pathogen specialists in the bacterial population, confirming that application of these biopesticides is a safe means of insect control. Populations of *B. thuringiensis* were transient on plant material, suggesting that selective pressure for resistance can be similarly transient. However, the genotype that dominates the natural community has been economically exploited more than any other, and selection for resistance to this strain may have occurred in natural populations of insects prior to the use of *B. thuringiensis* in pest control.

## Introduction

The *Bacillus cereus* group contains a number of clinically and economically important bacteria including the entomopathogen *Bacillus thuringiensis* (*Bt*); *Bacillus anthracis*, the causative agent of anthrax; and *B. cereus sensu stricto*, a species involved in potentially lethal food-poisoning as well as a wide range of opportunistic infections [1]. *Bt* is widely exploited in insect pest management. Its utility derives from the large quantities of proteinaceous toxins that form crystalline parasporal inclusions, termed the Cry (crystal) and Cyt (cytolytic) proteins [2]. The vast majority of commercially viable genetically modified (GM) insect resistant crops express one or more of these toxins and these crops covered approximately 46 million ha (worldwide) in 2008 [3]. *Bt* formulations are also the most successful organic microbial pesticide, with target hosts ranging from mosquitoes to Lepidopteran pests of agriculture, horticulture and forestry [4]. Although the structure and mode of action of *Bt* Cry toxins has been intensively studied, the biology and ecology of the bacterium is not fully characterized [1], [5]. Despite high levels of pathogenicity, the ability of *B. thuringiensis* strains to grow and sporulate effectively within insect cadavers is variable [6], [7]. Reports of natural outbreaks or epizootics of *Bt* are very rare in the field [8] and effective transmission of *Bt* between larvae has been difficult to demonstrate experimentally [9] and can require a high density of hosts and/or cannibalism [10].

Although it has been challenging to demonstrate how *Bt* is transmitted between hosts in the field, this bacterium is readily isolated from soil and plant material [11], [12], [13], [14], [15]. The variable success of *Bt* as a pathogen, the abundance of this bacterium in soil and plant material, and the reported lack of correlation between host abundance and the abundance of entomopathogenic *Bt* [14] remains puzzling and has also led to wide speculation on the ecological niche of *Bt*. It has been suggested that *Bt* is a soil micro-organism with incidental insecticidal activity [14]; that *Bt* is part of the phylloplane microbiota and has evolved to provide symbiotic protection against insect attack [15], [16]; or that *Bt* may be part of the commensal gut microbiota of many insects without causing overt disease [1]. With the exception of a quantitative examination of the claim that *Bt* can reproduce as a commensal [5] each of these hypotheses remain largely untested.

Improved understanding of the ecology of *Bt* and the dominant forces shaping the population structure of the *B. cereus* group will help address key issues for the ongoing exploitation of *Bt* such as: (1) managing the potential for widespread resistance to microbial sprays and GM crops [17]; (2) understanding the selective forces maintaining insecticidal toxin expression in natural populations; and (3) establishing whether insecticidal *Bt* is ecologically distinct from *B. cereus* strains that are capable of infecting humans [18]. Our aims, therefore, were to understand if *Bt* behaves as a true entomopathogen in the field by exploring the effect of the presence of an insect host on the abundance and population structure of the *B. cereus* group; to investigate how the application of live microbial pesticides might alter the indigenous bacterial population structure and thereby affect selection for the evolution of resistance to *Bt*; and finally to understand the ecology and phylogeny of *Bt* in comparison to that of its close relatives in the *B. cereus* group. In other words, are environmental and entomopathogenic strains from a single community ecologically distinct? And do environmental conditions that promote the proliferation of *Bt* also promote the proliferation of other members of this group?

In order to examine how environmental conditions are involved in generating and maintaining population structure within the *B. cereus* group we used a novel combination of experimental ecology and multi-locus sequence typing (MLST). MLST is an increasingly popular genotyping technique based upon the nucleotide sequences of loci within several housekeeping genes. For each locus, unique sequences are assigned unique allele numbers, and each specific allelic combination is assigned a sequence type (ST) [19], [20]. An MLST scheme has been developed for the *B. cereus* group and has been used to study the phylogenetic relationships and population structure of these bacteria [21], [22], [23], [24]. Although MLST has been applied to hundreds of *Bacillus* isolates (http://pubmlst.org/bcereus/) to our knowledge, no study has applied this technique in combination with the experimental manipulation of the *Bacillus* population or its hosts.

In this study we have explored the ecology of this group of organisms in relation to an insect host, the larvae of *Plutella xylostella* (the diamondback moth). A manipulative field trial was used to assess how bacterial abundance and population structure would be affected by the presence or exclusion of host insects and by the application of a commercial *Bt* biopesticide. MLST of strains collected during the trial was used to examine treatment effects upon the population genetic structure. All strains were phenotypically scored for the presence of entomocidal Cry toxins. Alleles and STs were assigned to habitat specific clades, based on genealogical reconstructions using CLONALFRAME [23], and the proportions of these alleles and STs amongst strains of each treatment group were compared. We have shown how insect hosts and biopesticide application can increase the *B. cereus sensu lato* population and increased the representation of entomocidal strains within this population. We found strong evidence for ecological differentiation between *B. cereus* clades both in terms of habitat association and the manner in which they responded to insect hosts. In contrast to previous MLST studies of the *B. cereus* group, we found a near epidemic population structure with a single genotype dominating the community, this genotype proved better able to colonize leaf surfaces (even in the absence of hosts) than non-pathogenic relatives in clade 3 and a higher proportion of isolates with this genotype expressed Cry toxin genes in comparison with other *B. thuringiensis* genotypes.

## Results

### *B. cereus* group abundance on leaves and in soil

**Figure 1:**
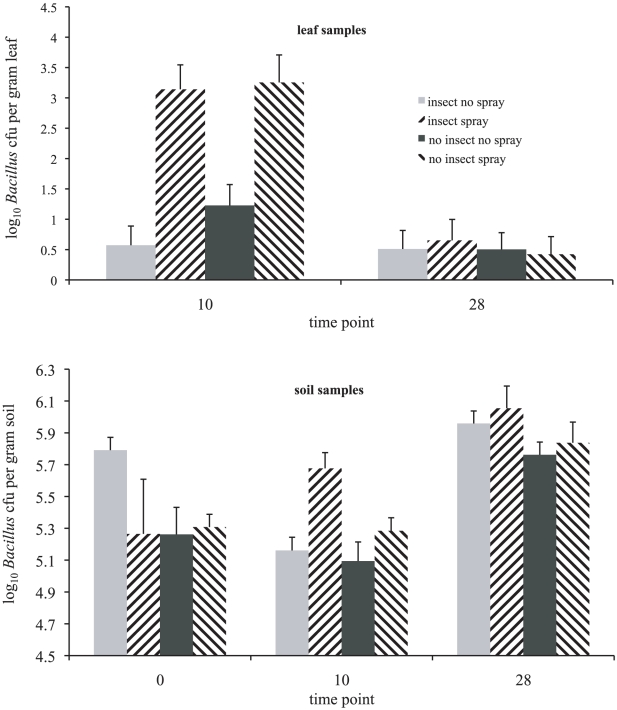
The effect of the presence of insect hosts and biopesticide application on bacterial abundance of the *Bacillus cereus* group in experimental enclosures. Populations of host insects (*P. xylostella*) were established in host addition cages six weeks before the first sampling time point (time point 0). Soil samples were taken immediately before the application of a *B. t. kurstaki* biopesticidal spray (DiPel DF) (time point T0) and then at 10 and 28 days after spray application. Leaf samples were taken at time points T10 and T28 only. Treatment labels insect/no insect indicate the experimental exclusion of Lepidopteran hosts or the experimental addition of diamondback moth populations; the labels spray/no spray indicate the application or absence of biopesticide. The data are colony-forming units per gram of sampled material from leaf and soil samples (two samples per experimental cage, twenty four cages in all). Data are means ± SEs.

The density of *B. cereus* group bacteria on leaf material was increased by the application of a *Bt* biopesticide, ten days after spraying (Figure 1, mixed model ANOVA, time point*spray interaction, *df*  = 1, Likelihood ratio  = 25.5, *p*<0.0001). However, bacterial density in leaf samples was not affected by the addition of *P. xylostella* insect hosts to experimental enclosures (*df*  = 1, Likelihood ratio  = 0.315, *p* = 0.575), nor did the presence of these hosts interact with microbial pesticide application (*df  = *1, Likelihood ratio  = 0.661, *p* = 0.416). In contrast, both the presence of hosts and application of *Bt* biopesticide affected bacterial density in soil (Figure 1). Host addition increased bacterial densities in soil over the whole course of the experiment (*df  = *1, Likelihood ratio  = 7.77, *p* = 0.0053). *Bt* biopesticide application had only a transient effect on bacterial density in soil (spray*time point interaction, *df  = *2, Likelihood ratio  = 9.63, *p* = 0.008); post-hoc treatment comparisons confirmed that sprays had their strongest influence on bacterial density on the 10 day post-spraying time point (effect size 1.04, *t* = 3.83, *p* = 0.0002). A marginally significant three-way interaction also suggested that this temporary effect of spraying was strongest when insect hosts were present (three-way interaction, *df  = *2, Likelihood ratio  = 6.02 *p* = 0.049).

### CLONALFRAME genealogy

**Figure 2:**
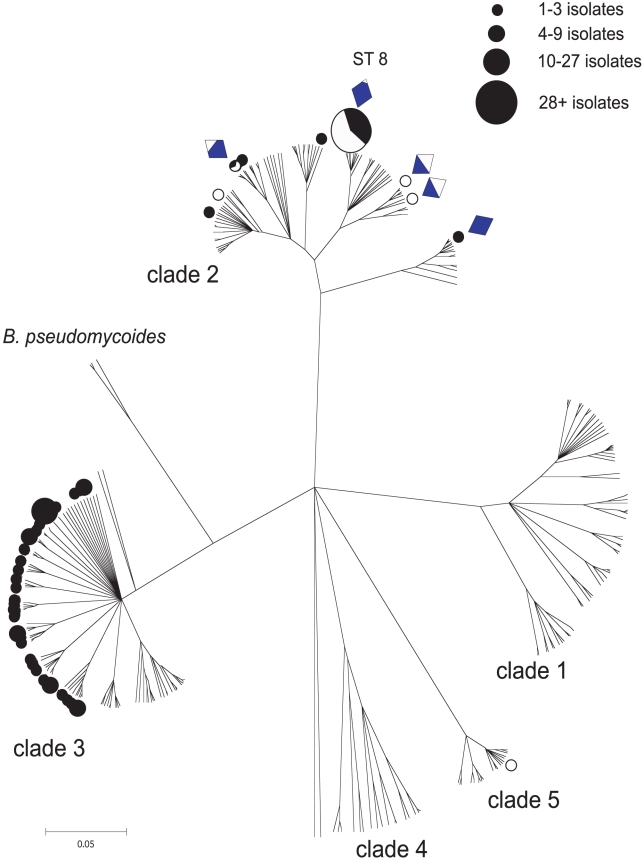
A CLONALFRAME genealogy of the *Bacillus cereus* group indicating the niche/habitat associations of STs recovered in this study. Sequence types (STs) isolated in this study have been mapped onto the genealogical tree with circular symbols, the remaining unmarked STs were recovered from the pubMLST isolates database. Filled black circles indicate STs were recovered from soil in this field study; open circles were recovered from leaf material only. Partially filled circles represent STs that were recovered from both soil and leaf material; the extent of the black fill inside indicates the frequency of isolates recovered from soil. The size of the circular symbols also indicates the abundance of that ST within the field population. Diamond symbols indicate which STs expressed Cry toxin parasporal inclusions. The extent of the blue fill within each diamond indicates the frequency of Cry-producing isolates within that ST in the field. The dominant sequence type (ST8) is also the genotype of the *Bt* strain (Dipel – *Btk* HD-1) that was used to spray experimental enclosures.

**Figure 3:**
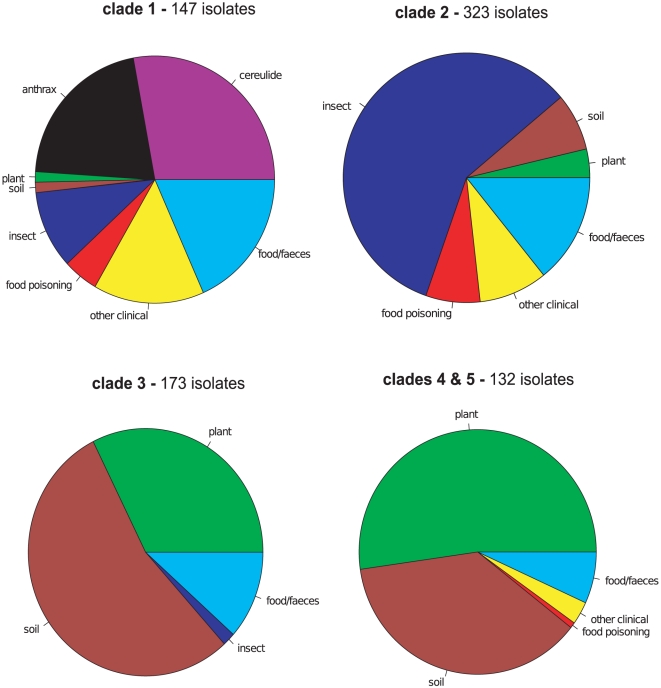
Clade level ecological differentiation of all *Bacillus cereus* group sequence types (STs) in the pubMLST isolates database. These charts show the frequency of sequence types within each clade that have a particular host associated niche or have been recovered from a particular habitat. Data for 298 STs and 773 isolates were recovered from this study, the pubMLST database and the MLST literature [21], [22], [25], [58] with 59, 94 and 106 STs from clades 1–3 respectively and 39 STs from both clades 4 and 5. Ecological niche was initially defined by the possession of ecologically significant plasmids: pX01 pX02 define *anthracis*; Cry toxin expression defines a strain as having an insect host and STs carrying the cereulide emetic toxin plasmid are also denoted as such. If this information was not available STs were defined according to clinical symptoms with which they were associated or the habitat from which they were isolated. STs were designated as of faecal origin rather than associated with food poisoning when there was no clear documentation of disease symptoms in hosts. If multiple isolates with a single ST had more than one ecological association this ST divided its contribution to the ecological categories in proportion to the frequency of isolates associated with each habitat/host, the sum of all these contributions being 1.

A CLONALFRAME analysis of the pubMLST isolates database and our own isolates resolved three major clades, as found in previous analyses [22], [24], [25]. The inclusion of isolates not incorporated in previous analyses revealed a deep branching genetic structure indicating the presence of additional, less populous, clades (Clades 4 & 5, Figure 2). The names of clades 1–3 follow previous studies: clade 1 contains *B. anthracis*, *B. cereus* emetic strains and other clinical isolates as well as approximately one third of the *B. thuringiensis* strains (Figure 3). Clade 2 contains the majority of *B. thuringiensis* isolates as well as a large number of isolates involved in enterotoxin associated food poisoning and septicaemia, and two isolates of *Bacillus mycoides* (Figures 2 & 3). Clade 3 contains all the strains designated as *Bacillus weihenstephanensis* in the database as well as three STs identified as Cry producing *thuringiensis* (STs 190 196, 200; Figure 3) and two isolates of *B. mycoides*; the remaining *B. mycoides* and two isolates of *Bacillus pseuodomycoides* were placed in a distinct subgroup of clade 3 (Figure 2). Clades 4 and 5 contain predominantly isolates of environmental origin collected from the Silwood Park campus of Imperial College, Ascot UK [26] as well as a small number of clinical isolates, some of which were previously associated with clade 2 (STs 101 and 111; Figure 3) [25]. All except one of the isolates recovered in this study were placed within clade 2 or clade 3, the remaining isolate mapped to clade 5.

The sequence type of the experimentally applied microbial pesticide is ST8 (Figure 2) and it is therefore unsurprising that the clade 2 isolates were dominated by ST8. However, ST8 was the commonest genotype in unsprayed plots and in the soil prior to the application of any biopesticide to the site and there was no prior use of *Bt* biopesticides on the farm on which the study was located. Thus, while we recovered 112 isolates with the ST8 genotype, 61 of these were recovered either before spraying with biopesticidal *Bt* (in T0 soil samples) or from unsprayed enclosures, indicating that this genotype dominated the natural community. Of the other STs recovered from clade 2, two have been identified previously as *thuringiensis* isolates (ST16 and ST18). Both ST16 and 18, and additional STs within clade 2 have been isolated as *B. cereus* in human infections, either from cases of diarrheal food poisoning (ST24) or from infections in immune-suppressed patients (ST18, 24, 166). We found both Cry toxin positive and Cry null (phenotypic *B. cereus*) isolates of ST8, ST18, ST24 and ST166 (Figure 2). The characteristics of all isolates with novel STs have been detailed in the online supporting information (Text S1).

### The effect of experimental treatments on the population structure of the *B. cereus* group

We hypothesized that if *Bt* reproduces in agricultural environments as an entomopathogen we should be able to detect the signature of this proliferation in the altered population genetic structure of the *B. cereus* group in response to the experimental addition of hosts and or biopesticidal sprays. With a single exception all leaf-isolated bacteria in this study mapped to clade 2, as did all Cry toxin production (Figure 2). These patterns suggested that entomopathogens are predominantly restricted to clade 2, although not all strains in clade 2 are necessarily entomopathogens. The remaining STs were primarily located within the *weihenstephanensis* clade (clade 3), which are typically soil bacteria [27] (Figure 3).

We tested whether our experimental treatments had altered the representation of entomopathogens in the natural population by using both an allele-based analysis and an isolate based analysis to investigate complementary hypotheses. The allele-based analysis used a genotypic definition of entomopathogenicity based on MLST variation, and allows us to test whether the entomopathogenic niche is associated with particular chromosomal genotypes. In contrast, the isolate-based analysis used a morphological definition of pathogenicity (Cry toxin production) that is associated with possession of virulence plasmids. Thus these two complementary analyses allow us to explore whether chromosomally linked traits and/or potentially mobile elements are important for the exploitation of insect hosts in the field.

### Allele based analysis of population structure

**Figure 4:**
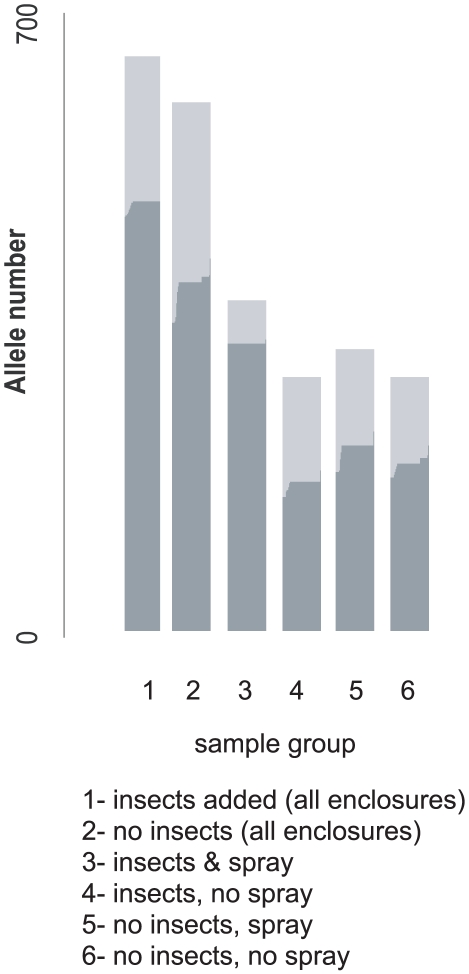
The allelic ancestry (entomopathogenic or non-pathogenic) of samples from experimentally defined isolate groups. Alleles from STs isolated from leaf samples were defined as being of pathogenic origin – these were predominantly from clade 2. All other alleles, those associated with STs that were only recovered from soil (predominantly clade 3) were defined as non-pathogenic. Ancestry was inferred using a no-admixture model in STRUCTURE, and is given for alleles associated with putatively pathogenic (dark grey) and non-pathogenic (light grey) genotypes. A CLONALFRAME genealogy was used to define background population structure (POPFLAG).

An allele-based analysis of our MLST-characterized strains was conducted that incorporated the possibility of allele exchange between strains. We defined strains based upon sample origin (leaf or soil): the alleles of isolates that had been recovered from leaf material were defined as having a pathogenic origin, and the alleles of all isolates that were only recovered from soil were defined as non-pathogenic. A habitat associated genotypic definition allows us to test the hypothesis that chromosomal traits associated with proliferation and/or colonization on leaf surfaces are important for the success of *B. cereus* group entomopathogens as well as allowing us to test the hypothesis that growth within hosts is more significant on a population level than epiphytic proliferation in the absence of insect hosts. A structure analysis tested how the presence of host insects and biopesticidal sprays affected the proportional representation of putative entomopathogens in the population using 104 isolates from time point T10 and 84 isolates from time point T28. The structure analysis indicated that a greater proportion of alleles with a pathogenic origin (leaf habitat associated alleles) were recovered from the enclosures with insect hosts (74% of alleles) than from enclosures without insect hosts (65% of alleles; Figure 4). Spraying with *Bt* biopesticide increased the proportion of alleles originating from putatively pathogenic strains, but only when insects were present in experimental enclosures, 87% of alleles originated from pathogenic STs compared to 58% in unsprayed plots. Where insects were absent from experimental plots the effect of spraying was negated: the probabilities of alleles having a pathogenic origin were 65% and 66% in sprayed and unsprayed plots respectively (Figure 4).

### Isolate-based analysis of population structure

In this analysis we defined entomopathogens as those strains that expressed the plasmid-borne Cry toxin parasporal inclusions. This allows us to test whether Cry toxin expression in the *B. cereus* group has a detectable fitness benefit in these bacteria in the field and in the presence of hosts. This analysis incorporated every isolate from time points T10 and T28 (a total of 301 isolates), all isolates were scored phenotypically by microscopic examination.

**Figure 5:**
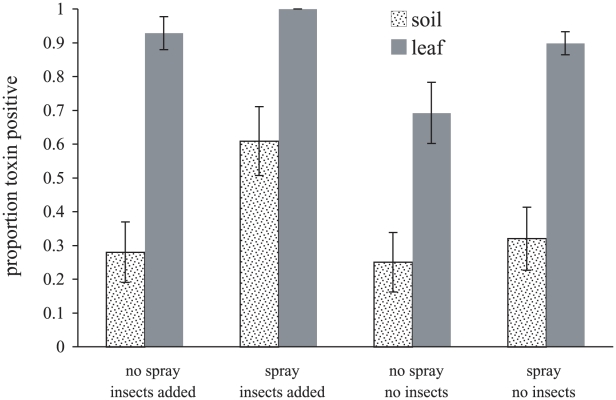
Habitat (sample origin) and experimental treatment affect the proportion of sampled *Bacillus cereus* isolates expressing Cry toxin parasporal inclusions. All the isolates collected during the 2006 field experiment were scored microscopically for the presence of proteinaceous parasporal inclusions (306 isolates in total). The data have been plotted according to sample origin (soil or leaf) and according to the treatments imposed upon experimental enclosures. Data are mean proportions for the six enclosures per treatment ±SEs.

The habitat from which isolates originated had a strong effect on the proportion of crystal producers in the population, those with leaf origin having a much greater probability of being Cry positive than soil derived isolates (*df*  = 1, χ^2^ = 103, *p*<0.0001; Figure 5). Biopesticide application and the addition of hosts both increased the proportion of crystal producing strains (*df*  = 1, χ^2^ = 12.2, *p*<0.0001; *df*  = 1, χ^2^ = 12.6, *p*<0.0001 respectively). Moreover, in this analysis, the effect of hosts did not depend upon spray application (spray*host interaction, *df*  = 1, χ^2^ = 0.85, *p* = 0.36; Figure 5).

### Exploring the association between bacterial clade and habitat

Mapping the sample origin of the field study isolates onto the genealogical tree revealed a strong correlation between clade identity and sample origin (Figure 2). The association of host/habitat with ST-based genotype was also investigated within the global *B. cereus* dataset (Figure 3). Whilst the soil was a universal reservoir for all isolates, only strains from clades 2 and 5 were isolated from leaf material (Figure 2). In contrast, within the global dataset there was a weaker correlation between clade and habitat/host association with STs. In the global dataset clades 1 and 2 in particular contain STs associated with both insect and vertebrate pathology. The restriction of clade 3 bacteria to the soil also does not hold in the wider data set, which includes isolates from a range of studies with different sampling regimes. An isolate based analysis of the pubMLST database and other sources does suggest a higher degree of clade-level specialization in the *B. cereus* group than this ST-based exploration of the data. However, the pubMLST database is not a natural population and an isolate-based analysis is potentially more subject to reporting and research biases than an exploration of the ecology of the recorded diversity at an ST level (Text S1). Both an ST based and isolate based analyses of genotype/ecology correlations have been included in the online supporting information for comparison (Text S1: Supporting Figure 1, Supporting Figure 2).

**Figure 6:**
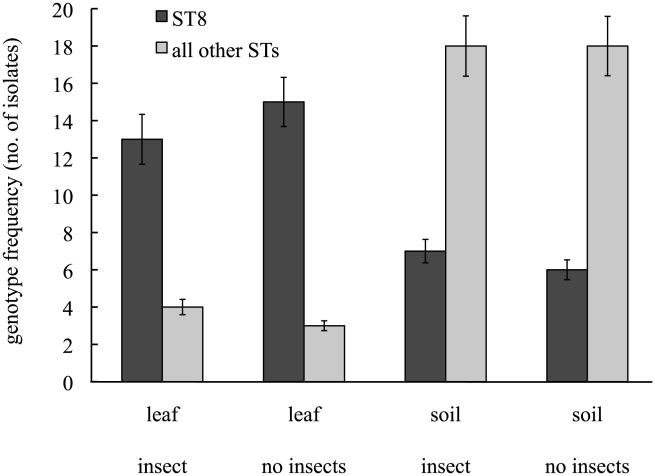
The association between habitat and genotype in unsprayed experimental enclosures naturally colonised by *Bacillus cereus* group bacteria. The dark bars represent the number of isolates identified as ST8, the light bars represent all other STs. Leaf and soil labels indicate sample origin; “insect” and “none” indicate the experimental addition or exclusion of host insects in experimental enclosures. Data are genotype frequencies ±SEs.

The association of isolates from clade 2 with plant material in this study was not, however, dependent upon the presence of insect hosts or the proliferation of bacteria within the hosts. This is best illustrated by examining the enclosures that did not receive biopesticidal sprays. A simple comparison of the proportion of isolates identified as ST8 (the commonest member of clade 2 in this study) shows that the putatively entomopathogenic ST8 was much more highly represented in the leaf samples than in the soil samples (χ^2^ = 24.7, *df*  = 2, *p*<0.0001; Figure 6) while the presence of hosts had no effect on the proportion of isolates identified as ST8 in this subset (χ^2^ = 0.014, *df*  = 1, *p* = 0.91; Figure 6).

**Figure 7:**
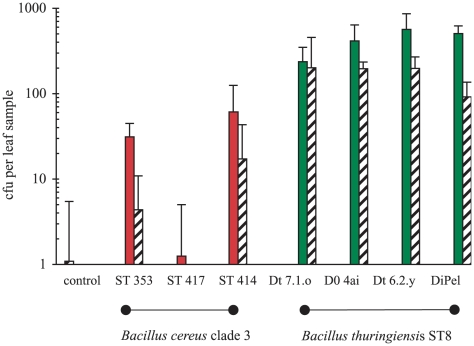
Variation in the ability of *B. cereus* clade 3 bacteria and *B. thuringiensis* (ST8) to colonize growing plants from experimentally inoculated soil. Cabbage seedlings (from surface sterilized seeds) were grown in autoclaved compost inoculated with either a clade 3 *B. cereus* strain; an ST8 isolate or in controls (without inoculation). Bacteria were recovered on *B. cereus* group selective media from leaf washes (epiphytic bacteria- solid bars) or homogenized surface sterilized leaf tissue (endophytic bacteria- cross hatched bars) after seedlings were at the 6-leaf stage. Data are the mean colony forming units (cfu) of *B. cereus* group bacteria per sample from four independent experimental replicates +SEs; no *B. cereus* group bacteria were recovered from leaf washes of control plants.

An additional greenhouse experiment showed that *B. thuringiensis* ST8 is much better than clade 3 *B. cereus* at colonizing the leaves of growing plants from experimentally inoculated soil. Plants were grown either in autoclaved compost (negative control); inoculated with a *Bt* ST8 isolate. Counts of bacteria in the *B. cereus* group in leaf samples from Chinese cabbage seedlings were measured using selective media containing polymyxin and a lecithinase indicator (egg yolk emulsion). There was at least an order of magnitude more *B. cereus* group bacteria in the leaf samples from the *Bt* ST8 treatment than in the *B. cereus* clade 3 treatment (epiphytic bacteria *t* = −6.11, *p*<0.0001; endophytic bacteria *t* = −4.96, *p* = 0.001; Figure 7). No *B. cereus* group bacteria were recovered from control plant leaf washes and there was a single colony recovered from the leaf homogenate (endophytic) control samples. The counts of bacteria in the *B. cereus* clade 3 treatment were not significantly different from the controls (epiphytic bacteria *t* = 1.25, *p = *0.222; endophytic bacteria *t* = 0.31, *p = *0.76). We also scored microscopically a subsample of *B. cereus* group isolates from the leaves of plants in *Bt* ST8 treatment - all 92 isolates were positive for the presence of parasporal crystalline inclusions, a phenotypic trait not shown by any of the *B. cereus* clade 3 strains.

## Discussion

Despite almost a century since its discovery we still know very little about the reproduction and field ecology of *Bt*, in part due to a lack of manipulative field experiments. In contrast to previous claims [14] we have shown that insect hosts can alter *B. cereus* group populations by both increasing population density and the proportion of strains expressing Cry toxins. Although we found no obvious *Bt-*killed cadavers on plant material and a transient population of *B. cereus/Bt* on the leaf surface, a moderate density of hosts (typically no more than 4–5 larvae per plant) nearly doubled the population of *B. cereus* group spores in soil. The rarity of cadavers on leaves, the transient low level population of *Bt* on plant surfaces [28], and the substantial accumulation of spores in soil suggests that opportunities for host-host transmission on leaves may be rare. Instead these results suggest a life cycle in which spores from cadavers, or cadavers themselves, quickly end up in soil with host-host transmission following on from a resting phase in a soil reservoir [29].

We performed MLST allele based and mobile element based (Cry toxin) analyses to explore how insect hosts in the field and biopesticide application altered population structure. We hypothesized that alleles linked to entomopathogenically important loci should increase in frequency in the population in response to the experimental addition of insect hosts. The MLST analysis showed that pathogenic alleles/STs only increase in frequency when both biopesticides and hosts are present. An analysis of Cry toxin carriage (with a larger sample size) found that hosts increase the frequency of toxin production independent of biopesticide spraying alone. Our overall interpretation is therefore that Cry toxins are beneficial to the *B. cereus* group when hosts are present and that biopesticides have a stronger impact on the bacterial community when they can proliferate within hosts. Nevertheless, MLST genotype and Cry toxin expression were tightly linked in this natural population and chromosomal genotype was strongly implicated in isolate ecology and in the ability of bacteria to colonize plant leaves. This association predominantly arose because one genotype ST8, was associated with most of the Cry toxin carriage and also dominated the leaf community, this high prevalence on leaves persisted in unsprayed experimental enclosures. We experimentally verified that several ST8 isolates have an improved ability to colonize growing seedlings from soil relative to non-pathogenic isolates in the *B. weihenstephanensis* dominated clade 3. This plant colonizing ability is independent of the possession of Cry toxin plasmids [30]. Other *Bacillus* spp. can colonize and adhere to leaf surfaces [31] and vegetative cells of *Bt, B. cereus* and *B. anthracis* have been found associated with plant roots [32], [33], [34]. The available evidence suggests that *Bt* and *B. anthracis* do not reproduce at a substantial level in soil or epiphytically, but that *Bt* can readily establish populations on leaves [4], [35], [36]. This study suggests that the movement of *Bt* from the soil reservoir to the aerial parts of plants, where susceptible hosts are present [4], is a key feature of its ecology. A similar transmission problem must also exist for *B. anthracis* which commonly infects ungulate hosts orally [37]. Therefore the association of *Bt* and *B. anthracis* with plants is probably not driven by the potential for epiphytic growth, but in order to increase the likelihood of infecting hosts.

A key aim of this study was to explore the ecology of *Bt* in relation to the selection pressure on Cry toxin resistance in natural populations. The poor persistence of high densities of *Bt* on plant foliage implies that relatively infrequent biopesticide applications will have little long-term effects on the *Bacillus* community. Elevated densities and changes in population structure effects were practically undetectable 28 days after spray application. More frequent *Bt* applications could have some impact on selection for resistance by altering the abundance or diversity of pathogens in the soil reservoir. The impact of changes in soil on selection pressure for resistance will be strongly mitigated by the very patchy natural distribution of *Bt* on plants. The real danger for the evolution of resistance arises if sprays are applied at a rate that results in a near continuous population of *Bt* on plants. We found that sprays in a temperate environment lend to high densities 10 days after application. Unsurprisingly, spray applications as frequent as every two or three days in low latitudes have led to multiple independent instances of the evolution of resistance to microbial pesticides in Lepidoptera on vegetable crops [38], [39].

The population structure of the natural *B. cereus* group community on plants also has implications for resistance evolution. This bacterial community was dominated by a single genotype (ST8) in agreement with work in the UK (R. Ellis unpubl. dat. available at http://www.pubmlst.org). This genotype is also used in the most successful biopesticidal sprays based on *B. t. kurstaki* HD-1 (DiPel). It produces predominantly two very similar Cry toxins (Cry1Ac, Cry1Ab) that are exploited in the majority of GM *Bt* corn and cotton varieties [40]. Several studies have found that a very high proportion of natural isolates carry Cry1Ac and Cry1Ab genes [41], [42]. Selection for resistance to naturally occurring toxins produced by ST8 may therefore have already elevated the frequency of resistance in insects prior to the use of *Bt* in pest management [43], [44]. The initial frequency of resistance to rarer toxin genes carried by other strains should be lower because of weaker past selection, and exploiting less common toxins may therefore have some benefits for resistance management.

This community-level study revealed a very tight relationship between bacterial clade and habitat/host association although this pattern is not repeated in the global *B. cereus* pubMLST dataset. On a global level, clades 1–3 have some differences in the proportion of isolates linked to particular hosts or disease symptoms (Figure 3) [22] but these generalizations do not hold well at a finer taxonomic resolution. Strains associated with insecticidal Cry toxins, pneumonia, septicaemia, and diarrheal food poisoning are widely distributed in both clades 1 and 2 [22] while clades 4 and 5 are almost as diverse. This poor correlation between pathogenicity and taxonomy in the global dataset results, at least in part, from the intrinsic mobility of many *B. cereus* virulence factors. Many major virulence factors are plasmid-borne in this group, *e.g. B. anthracis* pX01 and pX02 plasmids, the cereulide emetic toxin plasmid in *B. cereus* and the Cry toxin plasmids in *B. thuringiensis* [45], [46], [47]. Natural conjugation rates between *B. cereus* and *B. thuringiensis* strains within hosts can be extremely high (10^−1^ transconjugants per recipient) [48]. Chromosomal virulence factors are widely and variously distributed throughout the group [21] but may also have some horizontal mobility [49]. Nevertheless, occasional horizontal mobility should not preclude selection and recent clonal expansion producing a strong association between plasmid-associated phenotype and genotype at a fine geographical scale. Other studies using geographically coherent populations have also found associations between clade and Cry toxin gene expression [18], [27]. Sampling differences in different studies will also blur genotype habitat correlations in a global dataset. For example, many of the plant associated bacteria in clades 3–5 were recovered from a single study based on the plant *Rumex obtusifolius* [26]. This plant has a much lower growth habit than the *B. oleracea* used in this study. Mature *Rumex* leaves are close to the ground, are readily splashed with soil, and therefore carry more bacteria that are soil specialists. Thus, at a global level recombination, geographical divergence and diverse sampling regimes may be able to mask any local biological association between clade and ecology.

Evidence for dynamic Cry plasmid loss and gain was found in this study. Several STs were associated with isolates that either expressed or lacked Cry toxins and which are therefore formally *Bt* or *B. cereus* respectively. This is typical of the group: several of these STs have been previously identified as *Bt* (ST18 - *B. t. pakistani*; STs 56 and 57 - *B. t. darmstadiensis*); and described as *B. cereus* in wound infections (ST18, ST57) [22], [50] or diarrheal food poisoning (ST56); [25] and at least two additional genotypes (STs 15 and 109) show this dual identity [21], [22], [24], [51]. Clinical infections with *B. cereus* group bacteria have very rarely been linked to *Bt* [52], [53], [54]. This absence of recorded *Bt* infections in humans may have arisen because *B. cereus* clinical isolates are not routinely screened for Cry toxins or because real biological differences between Cry expressing and Cry null strains determine distinct ecological niches. Ecological differences could arise because certain genotypes have a much lower probability of carrying Cry plasmids or because regulatory cross-talk between plasmid and chromosome can affect virulence gene expression. Loss of the cereulide plasmid has been linked to a shift in site of infection type [25] and absence of Cry toxins has been associated with improved saprophytic growth in soil [55]. Ecological differentiation in the *B. cereus* group has been ascribed to changes in gene expression [49], and one unexplored possibility is that plasmids may be partly responsible for this variation.

This natural population of *Bt* and *B. cereus* was dominated by a single successful genotype [56]. Previous analyses of the global *B. cereus* database [22], food borne isolates [21], or clinical isolates [25], [57] have interpreted the *B. cereus* group as having a more reticulate population structure with more numerous or less dominant clonal complexes. Exceptions include the highly clonal nature of *B. anthracis* and the near clonal population structure of *B. cereus* emetic strains associated with cereulide poisoning [58]. Another study of an ecologically distinct population found that *B. cereus* clades 1 and 2 were both dominated by clonal complexes [27]. This discrepancy between data from unstructured sample collections and ecologically coherent studies may arise because the relative frequency of genotypes in a strain collection does not reliably represent their frequency in the field. Nevertheless, in our natural population, ST8 and its close relatives represent a highly dominant clonal complex of clade 2 strains in a manner strikingly similar to the way in which *B. anthracis* clones are prominent within clade 1. The analogy is not perfect: *Bt* ST8 may recombine more freely with relatives than *B. anthracis* and have more mobile plasmids, although *B. anthracis* virulence plasmids have now been found in other *B. cereus* group lineages [59]. Nevertheless, evidence is accumulating which suggests that the ST8 genotype is a successful specialist pathogen of Lepidoptera. Despite having the largest number of isolates in the pubMLST database (50 at this time) ST8 has never been associated with a clinical infection and is therefore strictly an invertebrate pathogen. In contrast to many sequence types, we found that a very high proportion of isolates were associated with Cry toxin expression; *B. anthracis* has a similar attachment to its virulence plasmids. One possible explanation for this pattern is that Cry toxin plasmids, despite their mobility, will not confer any long-term advantage to bacteria in the absence of unlinked genes that enable them to colonize leaves. Therefore, as we collect more data on the *B. cereus* group, the more the entomopathogenic specialists, such as ST8, resemble vertebrate specialists, such as *B. anthracis*, in terms of ecology and population genetics.

## Materials and Methods

### Field experiment

Twenty four fine wire mesh enclosures were each planted with six 4-week old Chinese cabbages (*Brassica pekinensis* var. “One Kilo SB”) in May 2006, in a field margin at Wytham Farm, Oxfordshire, UK. This farm has been managed primarily as pasture for at least three decades, a crop that is not typically sprayed with *Bt* based pesticides, and there has no been no recorded commercial use of these products at this site. We imposed two treatments in a balanced factorial experiment, using cage as a replicate. These treatments were addition/exclusion of a Lepidopoteran host and presence/absence of a *Bt-*based biopesticidal spray. The host addition enclosures were seeded with *Plutella xylostella* eggs and larvae in early June (50 eggs and 15 second instar larvae per plant) and with an additional 10 adults per cage in early July. Sprayed cages were inoculated with *B. t. kurstaki* HD-1 (DiPel DF, Valent Biosciences) on 20 July 2006 (T0) using 400 ml per cage from a stock of 40 g l^−1^ of formulated product.

Full sampling of the experiment took place 10 days and 28 days after sprays were applied. From each cage two independent soil samples and six independent leaf samples were collected; soil samples were approximately 1 g and were taken from the top 1–2 cm of the soil surface, leaf samples were approximately 2×2 cm and were taken from both emerging and fully mature cabbage leaves. Sampling methods and isolation of *B. cereus* group bacteria followed described protocols [26] except that all soil samples and leaf washes were pasteurized (65°C for 30 minutes) prior to plating out. All leaf and soil samples were weighed prior to processing. Two soil samples per cage were also taken immediately before spraying. A subset of randomly selected colonies (2 per soil sample or 6 per leaf) were streaked and stored as glycerol stocks. However, only independently sampled isolates (i.e. a single isolate from each sample tube) were included in the MLST analyses. In all, 384 samples were taken from the fully balanced experiment at time points 10 and 28 days post spraying (2 time points ×24 cages ×8 samples per cage) plus an additional 48 samples from the soil at time point 0. Only 288 independent isolates were available for the CLONALFRAME analysis as many leaf samples did not include any members of the *B. cereus* group. The MLST allele based analysis of experimental effects on population structure used only independent isolates from the fully balanced experiment in time points 10 and 28.

### Staining of parasporal inclusions

The crystalline parasporal inclusions of entomocidal Cry toxins produced by *Bt* were detected via oil-immersion microscopy. In brief, colonies were grown on Luria-Bertani (LB) agar plates until sporulation. After streaking on glass slides, bacteria were fixed in 100% methanol (10 minutes) and then stained with 0.05% w/v Coomassie Blue in a solution of 45% methanol (v/v) and 10% glacial acetic acid (v/v) for 20 minutes. Slides were quickly destained in a solution of 45% methanol (v/v) and 10% glacial acetic acid and washed in de-ionized water before inspection. Isolates were scored as potentially pathogenic if square or bipyrimidal blue stained inclusions were produced in sporulated cultures.

### Multi-locus sequence typing

Bacterial DNA was extracted using the CTAB method [60] and diluted to ∼150 ng/µl in nuclease-free H_2_O. PCR reactions containing 0.25 µM of each of the appropriate forward and reverse primers [24], 0.2 mM dNTPs (Bioline), 0.625 U Taq DNA Polymerase (Qiagen), 2.5 µl 10× buffer and 2 µl template DNA were prepared in a final volume of 25 µl. Reactions were carried out at the following conditions: initial denaturation at 94°C for 240 s followed by 10 cycles of 94°C for 30 s, 55°C for 30 s and 72°C for 80 s, followed by 15 cycles of 93°C for 30 s, 53°C for 30 s and 72°C for 80 s, followed by 25 cycles of 92°C for 30 s, 53°C for 30 s and 72°C for 80 s. A final extension period of 72°C for 480 s completed the reaction. Resulting DNA products were precipitated with 20% polyethylene glycolate solution and re-hydrated with nuclease-free H_2_O.

Sequencing reactions containing 0.2 µM of the appropriate forward or reverse primer, 0.33 µl Big Dye Terminator v3.1 mix (Applied Biosystems), 2 µl 5× buffer and 2 µl hydrated PCR product were prepared in a final volume of 10 µl. Cycling parameters were carried out as follows: initial denaturation of 96°C for 120 s followed by 38 cycles of 96°C for 20 s, 50°C for 10 s, 60°C for 240 s and a final extension of 72°C for 240 s. Sequencing products were precipitated with 5 M sodium acetate solution and subsequently separated and detected with an ABI Prism 3730 automated DNA sequencer (Applied Biosystems). The resulting sequence traces were aligned and edited using the Phineus software (http://www.phineus.org), and assigned allele numbers. Finally, each 7-locus allelic profile was assigned a sequence type.

### Leaf colonization experiment

In the autumn of 2009 we conducted a greenhouse leaf colonization experiment to compare the ability of *B. cereus* clade 3 bacteria and *Bt* ST 8 to colonize the leaf surfaces of Chinese cabbage seedlings (*B. pekinensis*) from soil. Seeds were surface sterilized in sodium hypochlorite [30] and planted in autoclaved soil based compost, with two seeds per 40 mm diameter pot. We set four negative controls (no inoculation); a treatment using three *B. cereus* clade 3 isolates (recovered from the 2006 field experiment) and a treatment with four *B. thuringiensis* ST8 isolates, each isolate being replicated four times. One ST8 isolate (D0 4ai) was recovered from the 2006 field experiment prior to spraying with DiPel, another isolate was derived from a stock of DiPel WP; two additional isolates were recovered from wild *Brassica oleracea* growing on sea cliffs in Dorset, UK [61]. Spores for soil inoculation were grown on *B. cereus* specific agar (Oxoid, UK), as per described protocols and enumerated using calibrated spectrophotometer readings. Soil was inoculated with 2×10^6^ spores g^−1^. Seedlings were cultivated in an insect-proof glasshouse with supplementary heating. After five weeks two leaf samples (200 mm^2^) were cut from each experimental pot. Epiphytic bacteria were recovered from plant surfaces using the same methods as in the field experiment [26]. Endophytic bacteria were recovered from homogenized surface sterilized samples [62]. *B. cereus* group bacteria were cultured from unpasteurized samples with *Bacillus cereus* specific agar.

### Data analysis

Analysis of bacterial densities used maximum likelihood mixed model ANOVA with enclosure as a random factor and spray, insect and time point as fixed factors. Significance testing was carried out via sequential deletion of terms from a full model in all analyses. For maximum likelihood models we have reported the degrees of freedom (df) of the model being tested as well the Likelihood ratio (the ratio of the likelihoods of statistical models in the test). Analysis of proportional data (STs, Cry toxin producers) used generalized linear modelling with binomial errors. The analysis of the leaf colonization experiment used a split-plot design and linear mixed effect modelling with bacterial isolate nested within block (fixed factors) and bacterial clade as a fixed factor. Model assumptions (normality, homoscedasticity, error distribution) were confirmed with graphical analyses. The above analyses were carried out in R (http://www.r-project.org).

### Genealogical analysis

A genealogy of the STs was produced using clonalframe, a model-based approach for determining bacterial microevolution [23]. This model determined the clonal relationships within the population with improved accuracy compared with standard phylogenetic inference techniques for recombining bacteria because it distinguished between point mutation and recombination - the two major sources of allelic polymorphisms. This model has been used successfully to distinguish species and subspecies clades within the *B. cereus* group [22]. Analysis was carried out on 228 isolates from the current study augmented with data from 286 unique STs from the *B. cereus* MLST database at http://www.pubmlst.org [63]; we specifically used data only from isolates associated with published papers in addition to isolates submitted by authors of this paper. A total of 315 unique STs were used to produce a genealogy of the entire *Bacillus* group. In each case, sequences of all 7 loci were concatenated and analyzed in 3 independent runs of clonalframe, each consisting of 100,000 iterations with the first 10,000 burn-in iterations discarded. Using the clonalframe tree comparison tool, described in the user guide, satisfactory convergence and mixing were confirmed using the Gelman–Rubin test [64], [65]. The consensus tree represents combined data from three independent runs with 75% consensus required for inference of relatedness.

### Probabilistic assignment of allele origin

The assignment of probability of potential origin of alleles to populations (pathogen/soil bacterium) was calculated for alleles from isolates within each sample group individually and the percentage of all isolates attributed to each origin population was determined as the sum of these probabilities. The hypothesis that different groups/treatments harbour different allele types was tested using the no admixture model in structure [66]. The clonalframe genealogy was used to define background population structure and individual alleles were independently assigned to source with a training set of alleles from pathogenic and non pathogenic strains distinguished from the test data with use of the usepopinfo flag. Probabilistic analysis in structure used 10,000 burn-in iterations and 10,000 subsequent iterations.

## Supporting Information

Text S1 A comparison of ST-based and isolate-based habitat-genotype correlations in the *B. cereus* group and a description of all isolates recovered in this study.
(0.31 MB DOC)
