# Supplementary material for: Environmental Factors Determining the Epidemiology and Population Genetic Structure of the Bacillus cereus Group in the Field
Source: PLoS Pathog. 2010 May 20;6(5):e1000905. doi: 10.1371/journal.ppat.1000905 (PMC2873914; doi:10.1371/journal.ppat.1000905)
Supplement: Text S1 — A comparison of ST-based and isolate-based habitat-genotype correlations in the B. cereus group and a description of all isolates recovered in this study. (0.31 MB DOC) [file ppat.1000905.s001.doc]

**Environmental factors determining the epidemiology and population genetic structure of the *Bacillus cereus* group in the field – online supporting information.**

**Genotype/host habitat correlations-introduction**

Presenting the relationship between clade or genotype involves a number of difficult issues. In the main manuscript we were primarily interested in describing the ecological association of each genotype rather than trying to use distributions of isolates in the database to make inferences about their ecology. The danger of the isolate based approach is that their numbers in the database are strongly biased by reporting rates and varying levels of research activity in different clades or environments. We therefore chose to present the available data on the host or habitat association of the *B. cereus* group at a sequence type level. Given that a range of isolates with a single ST can also have a range of habitat associations this meant dividing the contribution of each ST to several categories in some instances.

This ST based method also imposes another bias. A ST based description necessarily means that all STs contribute equally to the graphical analysis, thus rare STs that have only been isolated once from an opportunistic infection will contribute the same amount of data as for example ST8, which has 50 isolates in the pubMLST database and was recovered 61 times from unsprayed treatments in our field study. There is therefore an argument for assuming that high representation in the data set indicates some enhanced level of success in the *B. cereus* group as a whole. The habitat association data is also collected on a per isolate level and presenting this in a raw form without further manipulation, pre-existing biases notwithstanding, also has some merit.

For the above reason, and for the purpose of comparing ST-associated ecology and the isolate-level ecology we have presented both graphical analyses in this section. Definitions of habitat association follow those in the main paper, i.e. ecological niche was initially defined by the possession of ecologically significant plasmids: pX01 pX02 define *anthracis*; Cry toxin expression defines a strain as having an insect host and STs carrying the cereulide emetic toxin plasmid are also denoted as such. If this information was not available STs were defined according to clinical symptoms with which they were associated or the habitat from which they were isolated. Enterotoxin denotes *B. cereus* group strains that were involved in food poisoning but were not associated with cereulide production. STs were designated as of faecal origin rather than associated with food poisoning when strains were recovered from faeces but there was no clear documentation of disease symptoms in hosts. Isolate level data from our field study was also included into this analysis with the exception of the ST8 genotype in sprayed treatments.

The contingency table data for both isolates and STs have been displayed as mosaic plots using the vcd package in R v.2.6.2.

**Genotype/host habitat correlations-results**

The clearest difference between the two analyses is that the isolate by isolate display gives a stronger impression of clade by clade specialization (Supporting Figure 1). For example, both *B. anthracis* and cereulide producing *B. cereus,* contribute very few STs to the database, but contribute proportionally more data to the isolates database. Although this relatively high isolate number reflects the research interest in these potentially lethal pathogens these data do give an accurate impression of how virulence plasmid acquisition by these lineages has led to successful exploitation of distinct pathogenic niches. Similarly, the isolate analysis shows that at least 50% strains in clade 2 have been found associated with Cry plasmids, by far the largest contributor to that group is ST8 (60% of the insect associated isolates in clade 2 were ST8). Clades 3, 4, and 5 are also much more clearly dominated by environmental bacteria in the isolate analysis. The statistical analysis of the various groups’ contributions in Supporting Figure 2 also confirms that clade 1 has significantly more clinical isolates and STs; that clade 2 has significantly more insect and enterotoxin poisoning associated isolates and STs and that clades 3-5 have significantly more environmental isolates and STs. The major difference between the statistical analyses of the isolates and STs is quantitative rather than qualitative, the significance levels are typically higher in the isolate data (Supporting Figure 2)

This comparison between the isolate data and the ST data is also informative: the isolate analysis reflects the true rarity of enterotoxin associated food poisoning cases, whereas the fact that a relatively larger number of STs are associated with food poisoning supports the view that these are opportunitisic infections. The large number of STs associated with relatively few isolates in the plant and soil derived groups of clades 3, 4, and 5 suggest that these are by far the most diverse groups. The rapid discovery rate of novel STs from soil (mostly in clade 3) in this study and previous environmental studies (R. Ellis et al. unpubl. dat.) also confirms that there is much undiscovered diversity in soil bacteria. In contrast, the way in which so few STs dominated the serious clinical pathogens and the invertebrate pathogens suggests very few lineages have evolved to become specialist pathogens.

**Characteristics of novel STs discovered in the field experiment.**

These have been listed in Supporting Table 1.


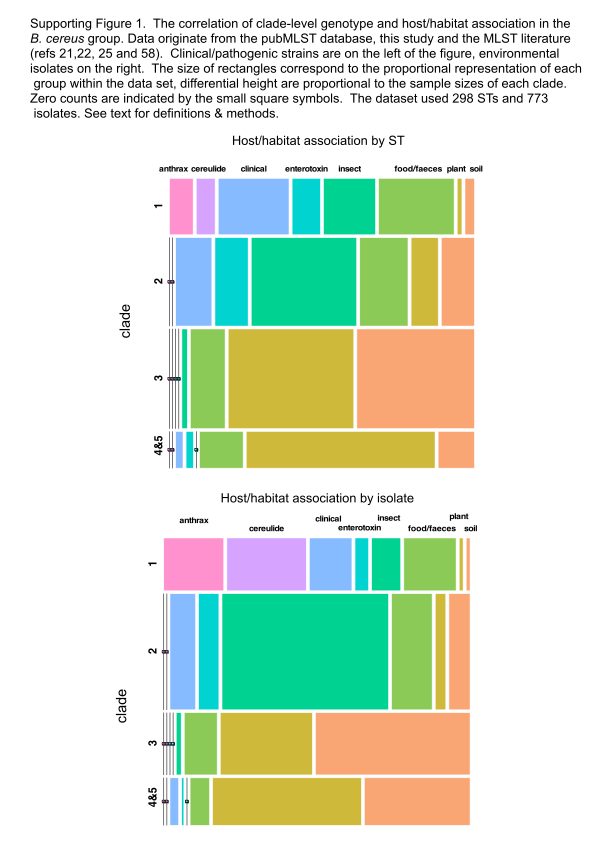


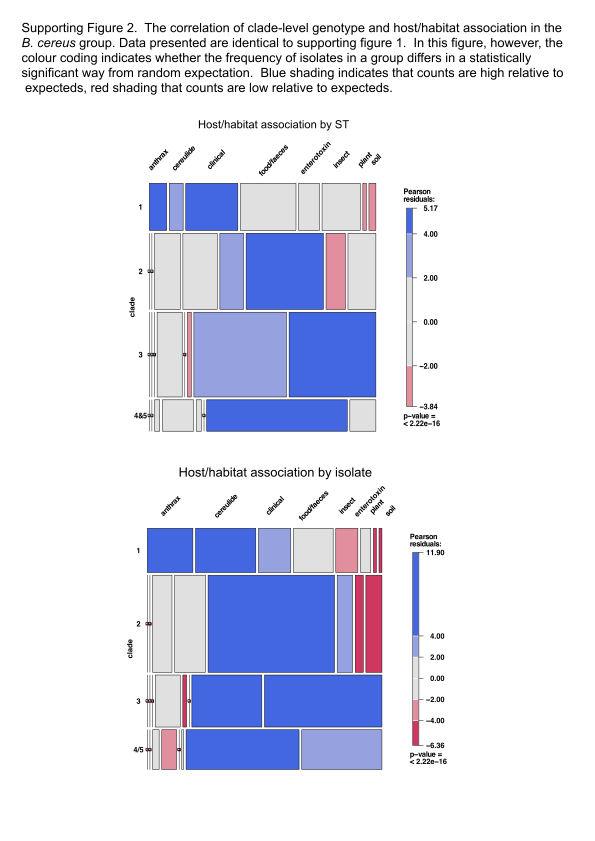


Supporting Table 1. Characteristics of isolates with novel sequence types recovered during the field experiment.

| **ST** | **Isolate** | **Source** | **Time point** | **Clade** | **Cry inclusion** | **Allele number** | | | | | | |
| --- | --- | --- | --- | --- | --- | --- | --- | --- | --- | --- | --- | --- |
|  |  |  |  |  |  | **glp** | **gmk** | **ilv** | **pta** | **pur** | **pyc** | **tpi** |
| 406 | 10ai | soil | T0 | 3 | none | 25 | 10 | 127 | 36 | 57 | 23 | 11 |
| 409 | 5bi | soil | T0 | 3 | none | 26 | 21 | 104 | 73 | 27 | 32 | 61 |
| 409 | 14ai | soil | T0 | 3 | none | 26 | 21 | 104 | 73 | 27 | 32 | 61 |
| 414 | 8bi | soil | T0 | 3 | none | 64 | 10 | 127 | 36 | 57 | 23 | 11 |
| 414 | 9bi | soil | T0 | 3 | none | 64 | 10 | 127 | 36 | 57 | 23 | 11 |
| 414 | 15ai | soil | T0 | 3 | none | 64 | 10 | 127 | 36 | 57 | 23 | 11 |
| 414 | 16bi | soil | T0 | 3 | none | 64 | 10 | 127 | 36 | 57 | 23 | 11 |
| 415 | 7bi | soil | T0 | 3 | none | 64 | 10 | 127 | 119 | 57 | 23 | 11 |
| 416 | 4bi | soil | T0 | 3 | none | 84 | 10 | 109 | 66 | 104 | 22 | 87 |
| 417 | 2ai | soil | T0 | 3 | none | 118 | 51 | 125 | 121 | 123 | 92 | 79 |
| 417 | 18bi | soil | T0 | 3 | none | 118 | 51 | 125 | 121 | 123 | 92 | 79 |
| 418 | 1bi | soil | T0 | 3 | none | 121 | 51 | 128 | 121 | 109 | 92 | 79 |
| 418 | 6bi | soil | T0 | 3 | none | 121 | 51 | 128 | 121 | 109 | 92 | 79 |
| 418 | 18ai | soil | T0 | 3 | none | 121 | 51 | 128 | 121 | 109 | 92 | 79 |
| 428 | 3bi | soil | T0 | 3 | none | 108 | 51 | 130 | 121 | 109 | 92 | 79 |
| 428 | 8ai | soil | T0 | 3 | none | 108 | 51 | 130 | 121 | 109 | 92 | 79 |
| 429 | 1ai | soil | T0 | 3 | none | 121 | 51 | 128 | 121 | 109 | 92 | 95 |
| 429 | 7ai | soil | T0 | 3 | none | 121 | 51 | 128 | 121 | 109 | 92 | 95 |
| 429 | 23bi | soil | T0 | 3 | none | 121 | 51 | 128 | 121 | 109 | 92 | 95 |
| 430 | 15bi | soil | T0 | 3 | none | 26 | 21 | 126 | 25 | 78 | 32 | 18 |
| 430 | 19bi | soil | T0 | 3 | none | 26 | 21 | 126 | 25 | 78 | 32 | 18 |
| 431 | 17ai | soil | T0 | 3 | none | 64 | 10 | 127 | 36 | 83 | 23 | 11 |
| 432 | 17bi | soil | T0 | 3 | none | 64 | 10 | 5 | 66 | 56 | 22 | 18 |
| 432 | 24bi | soil | T0 | 3 | none | 64 | 10 | 5 | 66 | 56 | 22 | 18 |
| 433 | 21ai | soil | T0 | 3 | none | 8 | 10 | 5 | 123 | 22 | 70 | 11 |
| 434 | 22ai | soil | T0 | 3 | none | 26 | 21 | 126 | 104 | 78 | 32 | 18 |
| 435 | 23ai | soil | T0 | 5 | none | 15 | 6 | 131 | 11 | 4 | 111 | 21 |
| 436 | 24ai | soil | T0 | 3 | none | 8 | 10 | 79 | 124 | 56 | 22 | 11 |
| 405 | 19.a.i | soil | T10 | 3 | none | 18 | 10 | 79 | 66 | 77 | 70 | 62 |
| 406 | 3.B.I | soil | T10 | 3 | none | 25 | 10 | 127 | 36 | 57 | 23 | 11 |
| 406 | 6.a.i | soil | T10 | 3 | none | 25 | 10 | 127 | 36 | 57 | 23 | 11 |
| 409 | 15.b.i | soil | T10 | 3 | none | 26 | 21 | 104 | 73 | 27 | 32 | 61 |
| 409 | 21.b.i | soil | T10 | 3 | none | 26 | 21 | 104 | 73 | 27 | 32 | 61 |
| 410 | 8.a.i | soil | T10 | 3 | none | 64 | 10 | 22 | 36 | 83 | 23 | 11 |
| 411 | 14.b.i | soil | T10 | 3 | none | 64 | 10 | 79 | 66 | 56 | 22 | 18 |
| 411 | 15.a.i | soil | T10 | 3 | none | 64 | 10 | 79 | 66 | 56 | 22 | 18 |
| 413 | 16.a.i | soil | T10 | 3 | none | 64 | 10 | 109 | 66 | 56 | 22 | 18 |
| 414 | 14.a.i | soil | T10 | 3 | none | 64 | 10 | 127 | 36 | 57 | 23 | 11 |
| 414 | 4.b.i | soil | T10 | 3 | none | 64 | 10 | 127 | 36 | 57 | 23 | 11 |
| 416 | 16.b.i | soil | T10 | 3 | none | 84 | 10 | 109 | 66 | 104 | 22 | 87 |
| 416 | 3.A.I | soil | T10 | 3 | none | 84 | 10 | 109 | 66 | 104 | 22 | 87 |
| 416 | 9.b.i | soil | T10 | 3 | none | 84 | 10 | 109 | 66 | 104 | 22 | 87 |

Supporting Table 1 cont.

| **ST** | **Isolate** | **Source** | **Time point** | **Clade** | **Cry inclusion** | **Allele number** | | | | | | |
| --- | --- | --- | --- | --- | --- | --- | --- | --- | --- | --- | --- | --- |
|  |  |  |  |  |  | **glp** | **gmk** | **ilv** | **pta** | **pur** | **pyc** | **tpi** |
| 416 | 13.b.i | soil | T10 | 3 | none | 84 | 10 | 109 | 66 | 104 | 22 | 87 |
| 416 | 4.a.i | soil | T10 | 3 | none | 84 | 10 | 109 | 66 | 104 | 22 | 87 |
| 417 | 2.A.I | soil | T10 | 3 | none | 118 | 51 | 125 | 121 | 123 | 92 | 79 |
| 419 | 19.b.i | soil | T10 | 3 | none | 26 | 21 | 126 | 104 | 27 | 110 | 61 |
| 422 | 8.b.i | soil | T10 | 2 | none | 119 | 8 | 40 | 30 | 2 | 109 | 93 |
| 422 | 10.b.i | soil | T10 | 2 | none | 119 | 8 | 40 | 30 | 2 | 109 | 93 |
| 423 | 12.a.i | soil | T10 | 3 | none | 120 | 63 | 5 | 15 | 124 | 70 | 94 |
| 426 | 12.b.i | soil | T10 | 3 | none | 25 | 10 | 22 | 122 | 106 | 23 | 11 |
| 427 | 12.o.1.3 | soil | T10 | 2 | none | 122 | 8 | 8 | 11 | 9 | 12 | 10 |
| 404 | 4Ai | soil | T28 | 3 | none | 8 | 10 | 5 | 123 | 57 | 70 | 11 |
| 405 | 16Ai | soil | T28 | 3 | none | 18 | 10 | 79 | 66 | 77 | 70 | 62 |
| 406 | 20Bi | soil | T28 | 3 | none | 25 | 10 | 127 | 36 | 57 | 23 | 11 |
| 406 | 23Ai | soil | T28 | 3 | none | 25 | 10 | 127 | 36 | 57 | 23 | 11 |
| 408 | 21Bi | soil | T28 | 3 | none | 26 | 10 | 109 | 66 | 56 | 22 | 18 |
| 411 | 1Bi | soil | T28 | 3 | none | 64 | 10 | 79 | 66 | 56 | 22 | 18 |
| 414 | 19Bi | soil | T28 | 3 | none | 64 | 10 | 127 | 36 | 57 | 23 | 11 |
| 414 | 3Bi | soil | T28 | 3 | none | 64 | 10 | 127 | 36 | 57 | 23 | 11 |
| 414 | 9Bi | soil | T28 | 3 | none | 64 | 10 | 127 | 36 | 57 | 23 | 11 |
| 414 | 17Ai | soil | T28 | 3 | none | 64 | 10 | 127 | 36 | 57 | 23 | 11 |
| 414 | 17Bi | soil | T28 | 3 | none | 64 | 10 | 127 | 36 | 57 | 23 | 11 |
| 414 | 8Bi | soil | T28 | 3 | none | 64 | 10 | 127 | 36 | 57 | 23 | 11 |
| 415 | 15Bi | soil | T28 | 3 | none | 64 | 10 | 127 | 119 | 57 | 23 | 11 |
| 416 | 6Bi | soil | T28 | 3 | none | 84 | 10 | 109 | 66 | 104 | 22 | 87 |
| 416 | 20Ai | soil | T28 | 3 | none | 84 | 10 | 109 | 66 | 104 | 22 | 87 |
| 418 | 15Ai | soil | T28 | 3 | none | 121 | 51 | 128 | 121 | 109 | 92 | 79 |
| 421 | 13Bi | soil | T28 | 3 | none | 64 | 10 | 127 | 36 | 26 | 23 | 11 |
| 425 | 16Bi | soil | T28 | 3 | none | 25 | 10 | 22 | 122 | 26 | 23 | 11 |
